# Detection of diverse genotypes of Methicillin-resistant *Staphylococcus aureus* from hospital personnel and the environment in Armenia

**DOI:** 10.1186/s13756-017-0169-0

**Published:** 2017-02-07

**Authors:** Hermine V. Mkrtchyan, Zhen Xu, Maria Yacoub, Mary M. Ter-Stepanyan, Hayk D. Karapetyan, Angela M. Kearns, Ronald R. Cutler, Bruno Pichon, Armen Dz Hambardzumyan

**Affiliations:** 1 Queen Mary University of London, School of Biological and Chemical Sciences, Mile End Road, London, E1 4NS UK; 2 School of Health, Sport and Bioscience, University of East London, Water Lane, London, E15 4LZ UK; 3 Antimicrobial Resistance and Healthcare Associated Infections Reference Unit, Public Health England, National Infection Service, Colindale, London, UK; 4 Yerevan State Medical University, Yerevan, Armenia

**Keywords:** MRSA, SCC*mec*, *ACME*, Pandemic, Maternity ward

## Abstract

**Background:**

Methicillin-resistant *Staphylococcus aureus* (MRSA) is a public health concern internationally. Studies examining a range of cohorts have been reported from various regions of the world, but little is known about the molecular epidemiology of MRSA in Armenia.

**Methods:**

Between May and September 2013, twenty isolates of methicillin-resistant *Staphylococcus aureus* (MRSA; *mecA* positive) were recovered from hospital personnel (*n* = 10; 9 females, 1 male) and environmental sites (*n* = 10) in the maternity ward of one of the teaching hospitals in Armenia.

**Results:**

Multi-locus sequence type clonal complex (MLST-CC) assignments inferred from *spa* typing data revealed the majority belonged to 3 pandemic lineages of MRSA including: t008-CC8-SCC*mec*V (*n* = 10; 7 from personnel); t021-CC30-SCC*mec*IV (*n* = 5; all environmental); and t1523-CC45 (*n* = 2; 1 from personnel), one harboured SCC*mec*V the other was SCC*mec* non-typable. The remainder identified as belonging to genotype t364-CC182, both of which harboured a novel SCC*mec* cassette with *kdp*, *rif5*, *ccrB2* and *ccrC* detected by PCR (both from personnel); and t325-CC88-SCC*mec*IV (*n* = 1; environmental). All MRSA were negative for the Panton-Valentine Leukocidin (PVL) locus and three CC8 strains were positive for the arginine catabolic element (ACME).

**Conclusions:**

In this small study, we report for the first time of the occurrence of diverse MRSA genotypes belonging to both pandemic and more sporadic international clones in Armenia harbouring the smaller SCC*mec* types and/or ACME, both of which have been associated with strain fitness. Further surveillance is warranted to better understand the prevalence, clinical and molecular epidemiology of MRSA throughout Armenia.

## Background

Methicillin-resistant *Staphylococcus aureus* (MRSA) is a major pathogen responsible for a wide range of mild to life threatening infections and is estimated to affect more than 150,000 patients annually in the European Union with associated costs of EUR 380 million to healthcare settings [1].

Reports of MRSA from an expanding range of ecological niches (healthcare, community, livestock, wildlife, environmental sources, etc.) are a public health concern internationally. Diversity in MRSA genotypes and their prevalence in different geographic areas continue to increase [2–5]. Studies examining a broad range of cohorts have been reported from various regions of the world [6–9], but little is known of the situation in some areas. Although MRSA clones from some countries have been well characterized [8, 10], there are few published studies describing the situation in the former USSR (Russia, Georgia), and none from Armenia [11–13].

The Republic of Armenia (part of the former Soviet Union) has three million inhabitants, half of whom live in the capital of Yerevan. Armenia is a low-middle income country and, currently, no population surveillance is being carried out in patients entering the hospital with the symptoms of illness compatible with staphylococcal disease. In this study, we report for the first time the molecular characterisation of MRSA recovered from hospital personnel and the environment in a University teaching hospital in the Republic of Armenia (part of the former Soviet Union). These data provide evidence for the first time of the occurrence of pandemic and more sporadic international MRSA clones in Armenia that harbour the smaller SCC*mec* types generally associated with strain fitness.

## Methods

### Study protocol

As part of a pilot surveillance study to assess the distribution and prevalence of MRSA, 450 samples were taken from hospital personnel (*n* = 150) and environmental sites (*n* = 300) in the maternity ward of one of the teaching hospitals in Armenia between May 2013 and September 2013. For hospital-based personnel (doctors, nurses and theatre nurses), samples were taken from the nasal cavity. Environmental sites included taps, patient examination chairs, surgical tables, nurse laboratory coats, baby scales, door handles and telephones. All specimens were inoculated onto nutrient agar (Oxoid, Basingstoke, UK) and incubated aerobically at 37 °C for 24-48 hours.

### Identification of the bacterial isolates

Suspected *S. aureus* were initially identified using conventional methods, including growth on Mannitol Salt Agar (Oxoid Ltd, Basingstoke, UK), slide coagulase and latex agglutination testing (ProLab Diagnostics, Neston, UK). To identify possible MRSA, isolates were sub-cultured onto Chromogenic MRSA agar (Oxoid Ltd, Basingstoke, UK). Those which grew yielding characteristic blue colonies were identified further by penicillin-binding protein (PBP2') agglutination testing (Oxoid Ltd, Basingstoke, UK).

### Phenotypic and genotypic characterisation of MRSA

Presumptive MRSA were screened for susceptibility to 4 antimicrobial agents (penicillin, cefoxitin, erythromycin and gentamicin) by disk diffusion and assigned as susceptible, intermediate or resistant according to the recommendations of the Clinical and Laboratory Standard Institute (CLSI) [14].

Isolates were characterised by real-time PCR to confirm they were *S. aureus* (*nuc* positive) and to determine their *mecA*, *mecC* and *luk-PV* status, as described previously [15]. MRSA were characterised further by *spa* typing [16], and staphylococcal chromosomal cassette *mec* (SCC*mec*) typing [17]. S*pa* typing data were used to infer multi-locus sequence type clonal complex (MLST-CC) assignments by reference to the *spa* server (http://spa.ridom.de/mlst.shtml), MLST (http://saureus.mlst.net) and in-house (Public Health England) databases. All CC8 MRSA were screened by PCR for the presence of the ACME element [18].

## Results

Over a 5 month period (May – September 2013), *S. aureus* was recovered from a total of 65 samples including 32 of 150 (21.3%) hospital personnel and 33 of 300 (11%) environmental sites in the maternity ward of one of the teaching hospitals in Armenia. Twenty (30.8%) *S. aureus* identified as MRSA. Half of these (*n* = 10) were recovered from hospital personnel including 9 females and one male; the remainder were recovered from the environment (Table 1).

**Table 1:** Susceptibility profiles and molecular characterisation of MRSA recovered from hospital personnel and environmental sites in the maternity wards of one of the teaching hospitals in Armenia

No	Source	PG	FOX	GM	ERY	*Spa* type	Inferred MLST-CC	SCC*mec* types	*ACME*
1	E	R	R	S	I^a^	t021	30	IV	ND
24	E	R	R	I^a^	R	t021	30	IV	ND
26	E	R	R	S	S	t021	30	IV	ND
30	E	R	R	S	R	t021	30	IV	ND
203	P	R	R	S	I^a^	t364	182^a^	Novel^b^	ND
210	P	R	R	S	S	t364	182^a^	Novel^b^	ND
221	E	R	R	S	I^a^	t021	30	IV	ND
222	P	R	R	S	I^a^	t008	8	V	-
223	P	R	R	S	S	t008	8	V	+
226	P	R	R	S	S	t1523	45	V	ND
227	P	R	R	S	S	t008	8	V	-
229	E	R	R	S	S	t008	8	V	+
230	E	R	R	S	S	t1523	45	Non-typable	ND
231	E	R	R	S	S	t008	8	V	-
232	E	R	R	S	R	t008	8	V	+
233	E	R	R	S	S	t325	88	IV	ND
236	P	R	R	S	I	t008	8	V	-
238	P	R	R	S	S	t008	8	V	-
244	P	R	R	S	I	t008	8	V	-
245	P	R	R	S	S	t008	8	V	-

*P* personnel, *E* environment, *PG* penicillin, *FOX* cefoxitin, *GM* gentamicin, *ERY* erythromycin, *Spa* staphylococcal protein A, *MLST-CC* Multi-locus sequence type clonal complex, SCC*mec* staphylococcal cassette chromosome mec, *ACME* arginine catabolic mobile element, *ND* not determined, + positive, - negative, all isolates were *nuc* and *mecA* positive, and *luk-PV* negative

^a^Singletons that do not fall into a clonal complex (CC) described in the *S. aureus* database (Skramm et al., 2007)

^b^ *kdp*, *rif5*, *ccrB2* and *ccrC* detected by PCR

*R* resistant, *S* susceptible, ^a^ *I* intermediate resistance. Zone sizes for intermediate resistance were ^a^ERY 20-22 mm; ^a^Gen 14 mm (Cockerill FR, [14])

All MRSA were *nuc* and *mecA* positive; no *mecC*-MRSA were identified. All were resistant to β-lactams (penicillin and cefoxitin); three were also resistant to erythromycin and six showed intermediate resistance (zone sizes 20-22 mm); in addition, one MRSA showed intermediate resistance to gentamicin (zone size 14 mm) (Table 1).

Most (17; 85%) study isolates belonged to pandemic genotypes of MRSA (Table 1), specifically: t008-CC8-SCC*mec*V (*n* = 10); t021-CC30-SCC*mec*IV (*n* = 5); t1523-CC45, one of which harboured SCC*mec*V, the other was SCC*mec* non-typable. The remainder belonged to more sporadic international lineages including t364-CC182-harbouring a novel SCC*mec* cassette with *kdp*, *rif5*, *ccrB2* and *ccrC* detected by PCR (*n* = 2); and t325-CC88-SCC*mec*IV (*n* = 1). All MRSA were PVL negative; three CC8 strains were ACME positive (Table 1).

## Discussion

The spread of antimicrobial resistant clones such as MRSA not only in healthcare and community settings but also livestock and companion animals is a major public health concern world-wide. In developing countries, the broader public health impact is worrisome due to the extensive and uncontrolled use of antimicrobial agents [7].

The aim of this study was to evaluate the clonal diversity, virulence and antibiotic susceptibility profiles of MRSA recovered from personnel and the environment in a University teaching hospital in the Republic of Armenia*.*

Twenty (30.8%) out of 65 *S. aureus* isolates recovered in this study were identified as MRSA. Various MRSA clones have been described globally, including some from the post-soviet countries [11–13, 19–21], however, little is known about MRSA in Armenia. The international ST239 clone has been reported as being dominant in Krasnoyarsk, Vladivostock and Georgia [11–13]. During the course of this small scale study we did not find evidence of this clone. Nevertheless, other international lineages were identified and a marked genetic diversity was apparent. The CC8-V lineage was predominant (*n* = 10; 50%) and was identified in both human and environmental sources. Three of 10 CC8-V isolates were recovered from the hospital personnel, the remainder were from the environment, which may reflect cross-contamination between personnel and the environment (Table 1). All CC8-V isolates were resistant to penicillin and cefoxitin; four also showed non-susceptibility to erythromycin. Distinct from the pandemic CC8-IV MRSA lineage associated with both healthcare- and community-associated infections [22, 23], CC8-V MRSA have been reported more sporadically [24]. In contrast to most reports of CC8-MRSA from various regions of the world (including Russia and Europe) that encode SCC*mec*IV [13, 25–29], all CC8 isolates in our study encoded SCC*mec*V and 3 of 10 were ACME positive. Aside from the successful USA300 (CC8-IV) clone of CA-MRSA in North America, the ACME element has been identified in a limited number of MRSA genotypes including ST5-II, ST59-IV, ST97-V, ST1-IV, ST5-IV and ST239-III [23, 30]. Interestingly, CC8-V has been found sporadically in Australia and Africa [24] but, to our knowledge, this is the first report of ACME in this lineage. Of note, the first case of CA-MRSA infection in Portugal caused by an ST8, *spa* type t008 strain was recovered from a male of Armenian ethnicity [31]. However, the isolate encoded SCC*mec*IV which differs from the CC8-V isolates identified in this study.

The second most common lineage identified was CC30-IV. CC30 is a widely disseminated pandemic clone, that has been associated with HA-MRSA, CA-MRSA and LA-MRSA [32]. In this study all (*n* = 5) CC30-IV isolates were recovered from the environment (Table 1). Whilst the pandemic HA-MRSA lineage encodes SCC*mec*II (ST36-II; UK EMRSA-16 clone), PVL-negative CC30-IV MRSA strains have been reported in countries such as Ireland [33] and Australia [32].

Two isolates belonged to CC45, *spa* type t1523 one with SCC*mec*V, the other was SCC*mec* non-typable. CC45 has is predominantly been associated with SCC*mec* type IV, which is also known as Berlin Epidemic strain or USA 600 [32]. However, CC45-MRSA-V strains have been reported in Germany, Australia and Portugal [32, 34]. Two isolates belonged to CC182; this clonal complex has been reported as a singleton [35] and MRSA belonging to CC182 have occasionally been identified in the UK and the Netherlands (http://spa.ridom.de/index.shtml).

In the current study we also identified a single CC88-IV strain. The CC88 lineage is prevalent among MRSA isolates from Africa [36] but has also been reported in Australia, Germany [32] the Netherlands, Portugal, Angola and Japan [33]. Interestingly, CC45-t1523 isolates (*n* = 2) were recovered from both hospital personnel and the environment, whereas CC182-t364 were isolated from the personnel only and CC88-t325 were identified from the environment only (Table 1). All 20 MRSA in our study were PVL-negative. This is consistent with the observations of other workers that PVL-positive MRSA is less prevalent in Europe than in the USA [32, 37]. As there are large Armenian communities in the US, Europe and Russia with relevant family links in Armenia it seems plausible that these clones were imported into Armenia from abroad in parallel with exchange of mobile genetic elements within the staphylococcal gene pool such as SCC*mec* and ACME.

## Conclusions

There are clear limitations in this small scale study. Clinical and epidemiological data were lacking; it is unclear whether any of the isolates may have been outbreak related or there were underlying risk factors such as previous healthcare contact or travel abroad. Similarly, we do not know if any isolates were multiply resistant as only a limited range of susceptibilities were determined. Nevertheless, this study provides insights into a previously unrecognised diversity of MRSA clones in Armenia including pandemic and more sporadic lineages seen internationally. These data also provide evidence that MRSA with a community-like genotype may be infiltrating healthcare settings in this country. In low and middle income countries healthcare- and community-associated infections are more challenging due to the lack of effective antimicrobial stewardship allied to infection control and prevention programmes [38]. Currently, no MRSA infection control programme exists in Armenia and no formal surveillance is being carried out in patients admitted to hospital with recognised risk factors, signs or symptoms compatible with MRSA/staphylococcal disease. Additional studies are warranted to further our understanding of the prevalence and molecular epidemiology of MRSA in healthcare settings in Armenia. In particular, we plan a more structured surveillance study of patients and hospital personnel with more detailed analyses to further our understanding of possible risk factors, burden of disease, genetic diversity and antimicrobial resistance rates to help inform national policy.

## Abbreviations

ACME: Arginine catabolic mobile element
CLSI: Clinical and Laboratory Standard Institute
ERY: Erythromycin
FOX: Cefoxitin
GM: Gentamicin
MLST-CC: Multi-locus sequence type clonal complex
MRSA: Methicillin-resistant *Staphylococcus aureus*
PG: Penicillin
PVL: Panton-Valentine Leukocidin
SCC*mec*: Staphylococcal cassette chromosome mec
